# Sp1 facilitates continued HSV-1 gene expression in the absence of key viral transactivators

**DOI:** 10.1128/mbio.03479-23

**Published:** 2024-02-13

**Authors:** Catherine N. Sodroski, Hyung Suk Oh, Shu-Fan Chou, David M. Knipe

**Affiliations:** 1 Department of Microbiology, Blavatnik Institute, Harvard Medical School, Boston, Massachusetts, USA; 2 Program in Virology, Harvard Medical School, Boston, Massachusetts, USA; Columbia University Medical Center, New York, New York, USA

**Keywords:** herpes simplex virus, transcription factor, interferon, transactivator, ICP0, Sp1, VP16

## Abstract

Productive replication of herpes simplex virus (HSV) relies upon a well-ordered transcriptional cascade flowing from immediate-early (IE) to early (E) to late (L) gene products. While several virus-encoded transcriptional activators are involved in this process, IE and E gene promoters also contain multiple binding sites for the ubiquitously expressed cellular transcription factor Sp1. Sp1 has been previously implicated in activating HSV-1 gene transcription downstream of these sites, but why Sp1-binding sites are maintained in the promoters of genes activated by virus-encoded activators remains unclear. We hypothesized that Sp1 enables continued HSV-1 transcription and replication when viral transactivators are limited. We used a depletion-based approach in human foreskin fibroblasts to investigate the specific contribution of Sp1 to the initiation and progression of the HSV-1 lytic gene cascade. We found that Sp1 increased viral transcript levels, protein expression, and replication following infection with VP16- or ICP0-deficient viruses but had little to no effect on rescued viruses or during wild-type (WT) HSV-1 infection. Moreover, Sp1 promoted WT virus transcription and replication following interferon treatment of fibroblasts and thus may contribute to viral immune evasion. Interestingly, we observed reduced expression of Sp1 and Sp1-family transcription factors in differentiated sensory neurons compared to undifferentiated cells, suggesting that reduced Sp1 levels may also contribute to HSV-1 latent infection. Overall, these findings indicate that Sp1 can promote HSV-1 gene expression in the absence of key viral transactivators; thus, HSV-1 may use Sp1 to maintain its gene expression and replication under adverse conditions.

**IMPORTANCE:**

Herpes simplex virus (HSV) is a common human pathogen that actively replicates in the epithelia but can persist for the lifetime of the infected host via a stable, latent infection in neurons. A key feature of the HSV replication cycle is a complex transcriptional program in which virus and host-cell factors coordinate to regulate expression of the viral gene products necessary for continued viral replication. Multiple binding sites for the cellular transcription factor Sp1 are located in the promoters of HSV-1 genes, but how Sp1 binding contributes to transcription and replication of wild-type virus is not fully understood. In this study, we identified a specific role for Sp1 in maintaining HSV-1 gene transcription under adverse conditions, as when virus-encoded transcriptional activators were absent or limited. Preservation of Sp1-binding sites in HSV-1 gene promoters may thus benefit the virus as it navigates diverse cell types and host-cell conditions during infection.

## INTRODUCTION

Herpes simplex virus 1 (HSV-1) infections are widespread, with a majority of the population estimated to be infected worldwide (1). While the primary symptoms of HSV-1 infection are orolabial lesions, clinically severe disease such as ocular keratitis and encephalitis can occur following infection, particularly in immunocompromised individuals (2). HSV-1 is a large, double-stranded DNA virus that transcribes and replicates its ~152-kilobase-pair genome within the nucleus of the infected host cell. HSV-1 gene transcription occurs in a sequential progression from IE to E to L gene products (3, 4). IE proteins enable the E proteins required for viral DNA synthesis to be expressed, and viral DNA replication and the ultimate production of infectious viral progeny are both dependent on IE and E gene transcription and expression. The lytic gene cascade is required for the production of new infectious virions and is relevant to both lytic infection in epithelial tissues and during reactivation from latently infected neurons (4, 5).

Coordination between virus-encoded and cellular proteins within the nucleus drives this lytic gene cascade forward. Cellular RNA polymerase II (RNA Pol II) is required for HSV-1 transcription, for example, and the viral tegument protein VP16 acts in conjunction with the cellular proteins HCF-1 and Oct-1 to coordinate promoter-specific activation of IE genes (4, 6–8). VP16 triggers the onset of the lytic gene cascade with IE gene transcription, but the subsequently expressed IE proteins ICP0 and ICP4 also enable efficient HSV-1 gene expression (9–12). ICP0 is a viral E3 ubiquitin ligase that targets restrictive host-cell proteins for degradation and promotes IE, E, and L gene expression (9, 10, 13–15). Both VP16 and ICP0 can promote the removal of nucleosomes and restrictive heterochromatin deposited on incoming viral genomes to increase viral DNA accessibility and subsequent gene expression (16–19). ICP4 is a viral transcription factor required for robust E and L gene expression (11, 20). ICP4 can bind nonspecifically or to specific sequences in viral promoters and can recruit the general RNA Pol II machinery to HSV-1 DNA to increase transcription of most viral genes (20–23).

HSV-1 thus encodes three transactivating proteins capable of stimulating and enhancing transcription at IE and E gene promoters. And yet, HSV-1 gene promoters include binding sites for host-cell as well as viral proteins (4). These include multiple GC-rich sequences, or GC boxes, in IE and E gene promoters that can be bound by the cellular transcription factor Sp1 (Specificity protein 1) (4, 24, 25). Sp1 is a ubiquitously expressed zinc-finger transcription factor with well-established roles regulating viral and cellular gene transcription (26, 27). Sp1 binding has been implicated in the transcriptional activation of specific IE and E gene promoters, and Sp1 binding has been detected at IE, E, and E/L gene promoters across the viral genome, with increased promoter binding observed prior to viral DNA replication (24, 25, 28).

Sp1 is therefore seemingly well positioned to contribute to viral gene expression within the infected-cell nucleus. However, prior studies demonstrated that Sp1-binding sites were not required for high levels of viral gene transcription and that the viral protein ICP4 could effectively substitute for the loss of Sp1-binding sites upstream of the E gene thymidine kinase (*tk*) promoter (29, 30). Why Sp1-binding sites are nevertheless maintained in viral gene promoters remains unclear. We hypothesized that, during HSV-1 infection, Sp1-binding sites may become relevant for continued viral gene expression and replication when transcriptional activation by virus-encoded proteins is absent or limited. In this study, we investigated the specific role of Sp1 in the regulation of viral gene transcription and characterized how Sp1 activity impacts HSV-1 infection in the absence of key viral transactivators.

## RESULTS

### Sp1 depletion does not significantly impact WT HSV-1 replication

**Fig 1:**
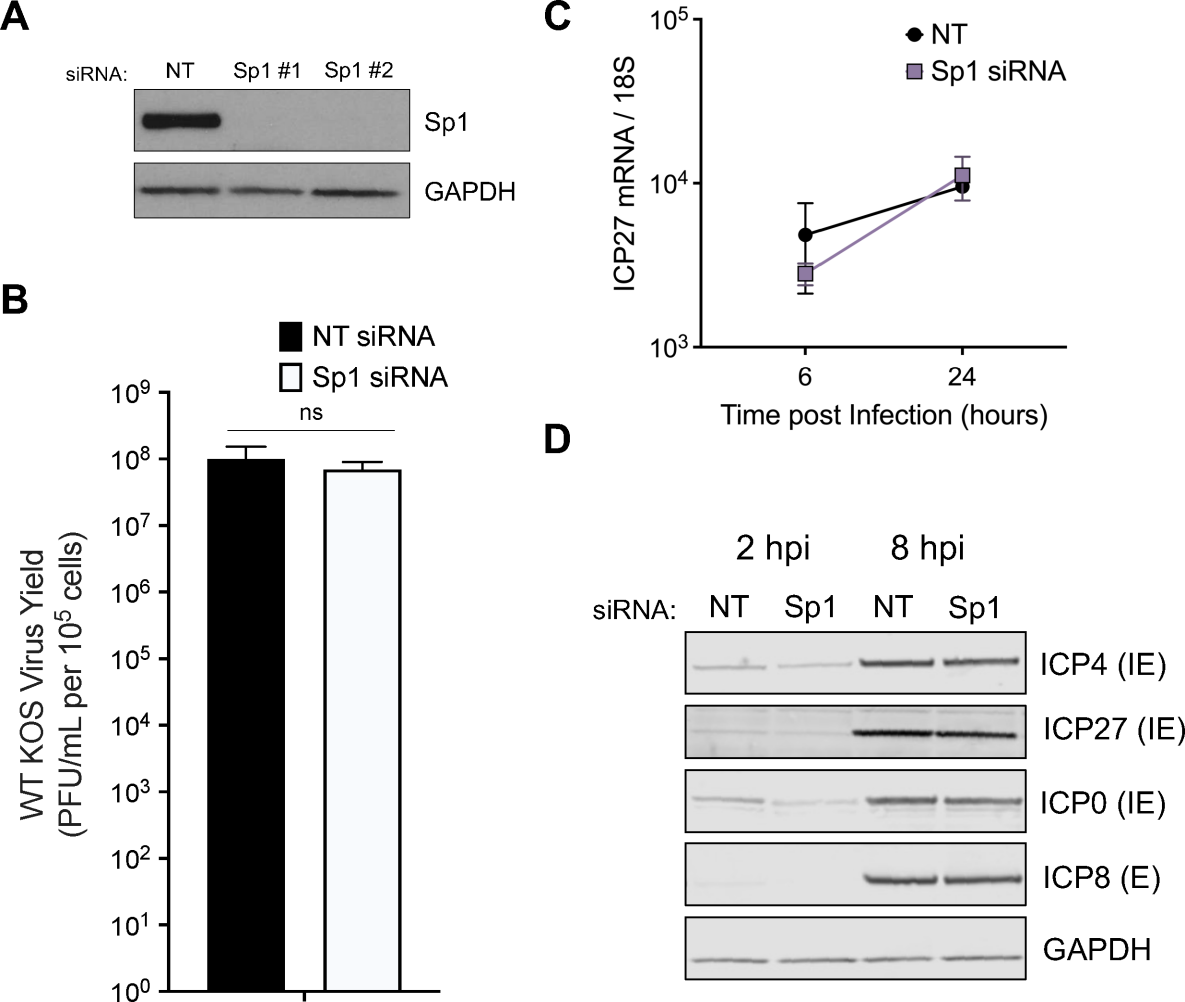
Sp1 does not significantly impact WT HSV-1 replication or gene expression. (**A**) Immunoblot analysis of Sp1 levels in HFFs following treatment with non-targeting (NT) control siRNAs or individual Sp1-specific siRNAs (Sp1 #1, Sp1 #2). (**B**) Viral yields at 24 h following infection of control or Sp1-depleted HFFs with the HSV-1 WT KOS strain (MOI = 1). (**C**) IE (*ICP27*) gene transcript levels were measured by qRT-PCR and compared in Sp1-depleted cells and control-treated HFFs following WT KOS infection (MOI = 1). (**D**) Immunoblot analysis of viral protein expression following WT virus infection of NT and Sp1 siRNA-treated HFFs (MOI = 3). Statistical significance in panels B and C was determined by two-way ANOVA followed by Tukey’s multiple comparison test. Mean ± S.D. (*error bars*), *P < 0.05, **P < 0.01.

To test the role of Sp1 during lytic HSV-1 infection, we used a depletion-based approach. We used individual Sp1-specific siRNAs to deplete Sp1 expression in human foreskin fibroblasts (HFFs) (Fig. 1A) and confirmed that cell growth was equivalent in untreated as well as non-targeting and Sp1-specific siRNA-treated fibroblasts (Fig. S1). To test the effect of Sp1 during WT virus lytic infection, we infected Sp1-depleted HFFs with the HSV-1 KOS strain. Surprisingly, we observed no significant effect of Sp1 depletion on total virus yield (Fig. 1B), with similar results obtained for each of the individual siRNAs (Fig. S2A and B). We probed the transcription and expression of several HSV-1 genes containing Sp1 sites in their promoter, including the IE genes ICP4 (31), ICP27 (32), and ICP0 (33), as well as the E gene ICP8 (34). There was no significant effect of Sp1 depletion on IE transcript levels following WT virus infection (Fig. 1C), and there was little to no change in IE or E protein expression observed at 8 h post infection (hpi) (Fig. 1D). However, while we observed no overall reduction in viral transcript or protein levels, we did observe slight reductions in both at the earlier time points measured following WT virus infection. These results indicated that Sp1 is potentially relevant to IE and E transcription during the initial stages of infection following viral entry into the nucleus. The virus-encoded transactivators VP16 and ICP0 are key drivers of transcription at this stage, and their activity during WT virus infection may mask any Sp1-mediated effects on viral transcript levels. Accordingly, we hypothesized that HSV-1 would be more sensitive to the levels of Sp1 expression in the absence of these virus-encoded transcriptional activators.

### Sp1 promotes viral gene expression in the absence of functional VP16

**Fig 2:**
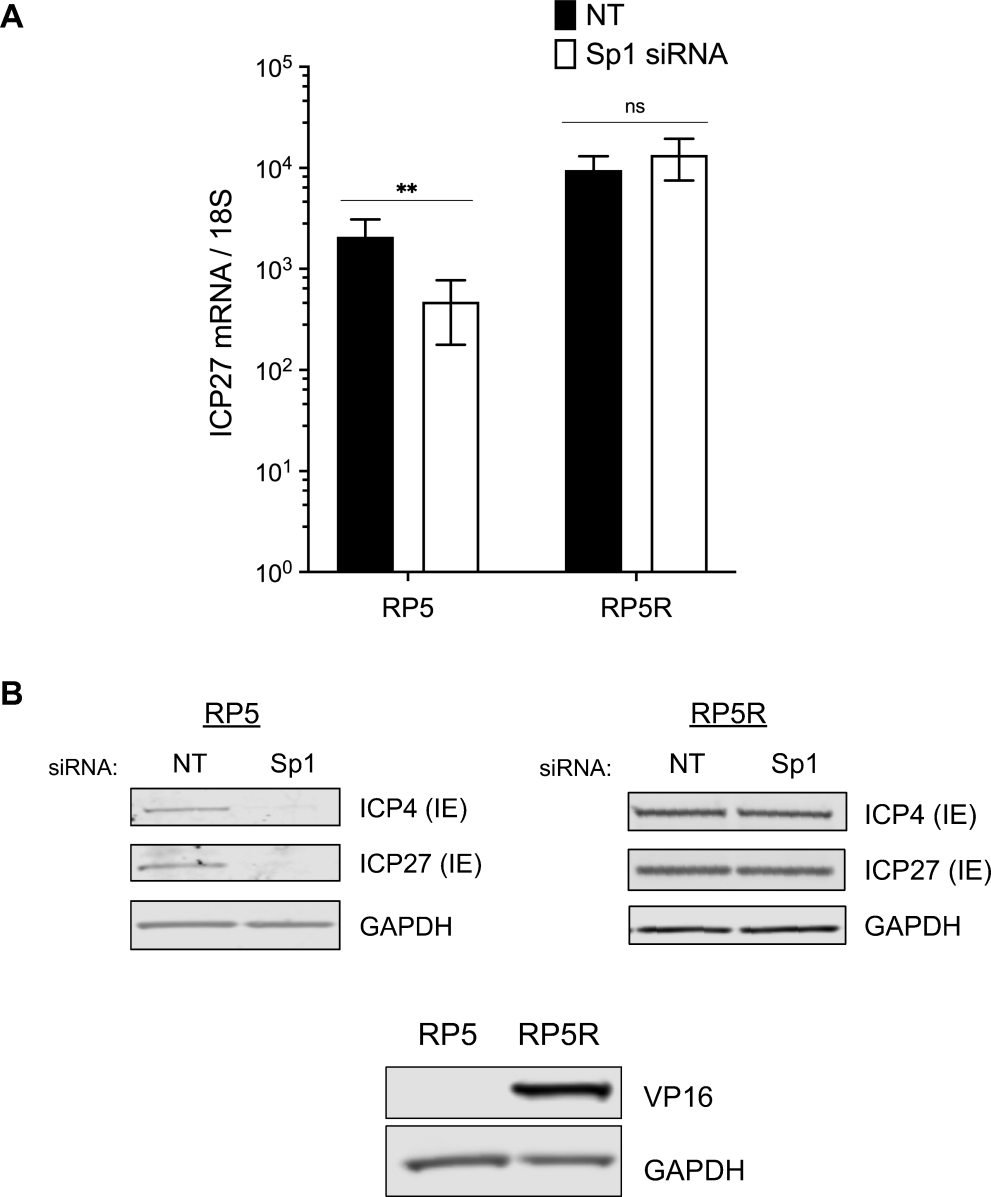
Sp1 promotes viral gene expression in the absence of functional VP16. (**A**) IE (*ICP27*) gene transcript levels assessed by RT-qPCR following infection of control or Sp1-depleted HFFs with the RP5 VP16-mutant virus or the rescued virus RP5R (MOI = 10, 6 hpi). (**B**) Immunoblot analysis of IE (ICP4, ICP27) and VP16 protein levels following RP5 or RP5R infection (MOI = 3, 24 hpi). Statistical significance in A determined by two-way ANOVA followed by Tukey’s multiple comparison test. Mean ± S.D. (*error bars*), *P < 0.05, **P < 0.01.

To first test the contribution of Sp1 to viral transcription in the absence of VP16, we infected Sp1-depleted HFFs with the HSV-1 RP5 mutant virus expressing a non-functional VP16 protein (35). Following RP5 virus infection, we observed a significant ~5-fold reduction in IE *ICP27* gene transcripts in the Sp1-depleted cells relative to control cells (Fig. 2A). ICP4 and ICP27 protein expression was similarly reduced and barely detectable in the Sp1-depleted cells compared to control cells (Fig. 2B). Sp1 depletion reduced viral transcript levels when functional VP16 was absent during RP5 infection, but Sp1 expression level had no impact on IE transcript or protein levels following infection with the rescued virus RP5R (Fig. 2A and B; Fig. S3). These results indicated that, when functional VP16 was present, Sp1 no longer contributed significantly to viral IE gene transcription. This finding was consistent with the results obtained above following WT virus infection. However, the results obtained following RP5 infection indicate that Sp1 may play a significant, if restricted, role during HSV-1 lytic infection in promoting virus gene transcription when virus-encoded transcriptional activators like VP16 are limited or absent.

### Sp1 promotes virus replication and gene expression when the IE protein ICP0 is absent

**Fig 3:**
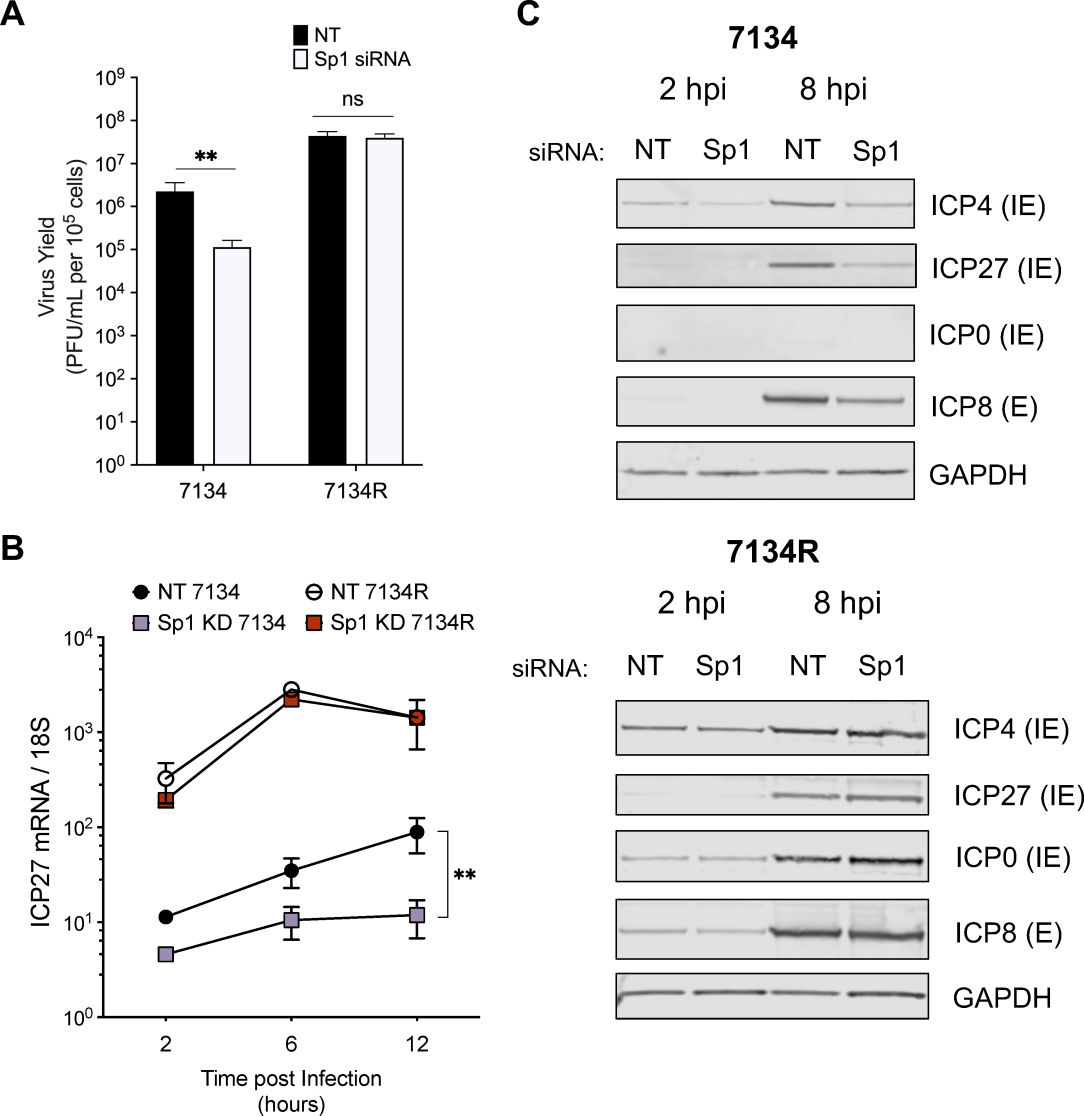
Sp1 depletion reduced replication of an ICP0-null virus. (**A**) Viral yields were determined following infection of NT-treated or Sp1 siRNA-treated HFFs with ICP0-null 7134 or ICP0-positive 7134R viruses (MOI = 1, 24 hpi). (**B**) RT-qPCR analysis of IE transcript levels following 7134 or 7134R virus infection (MOI = 1) in control or Sp1-depleted HFFs. (**C**) Immunoblot analysis of IE and E viral gene expression following Sp1 knockdown and 7134 or 7134R virus infection (MOI = 3) in HFFs. Statistical significance was determined in panels A and B by two-way ANOVA followed by Tukey’s multiple comparison test. Mean ± S.D. (*error bars*), *P < 0.05, **P < 0.01.

We next investigated whether Sp1 impacted HSV-1 replication in the absence of the IE transcriptional activator ICP0. Following infection with the ICP0-null virus 7134 and the ICP0-positive rescued virus 7134R (36), we observed that Sp1 depletion led to reduced ICP0-null virus yields but had no effect on 7134R replication (Fig. 3A; Fig. S4). In addition, during 7134 infection, we observed reduced *ICP27* IE gene transcript levels and reduced IE and E protein expression in Sp1-depleted fibroblasts (Fig. 3B and C). Sp1 depletion had little to no effect on total transcript or protein levels during 7134R infection although there was a slight decrease in viral transcript and protein levels observed at 2 hpi (Fig. 3C). This observed decrease at 2 hpi was consistent with the findings described above during WT HSV-1 infection and with an initial reduction in 7134R virus yields observed at 6 hpi in time course experiments and suggests that Sp1 may initially promote WT virus expression and replication but is subsequently redundant (Fig. S4C).

Overall, as observed following RP5 and RP5R infections, the expression of a virus-encoded transactivator and the resulting production of additional viral gene products could compensate effectively for Sp1 depletion. However, Sp1 did contribute to increased viral transcription, gene expression, and replication in the absence of functional VP16 or ICP0. Of note, during 7134 virus infection, VP16 is present and likely functional. Accordingly, the onset of viral gene transcription is not blocked as extensively during ICP0-null virus infection when only ICP0 transactivation is impaired. Nevertheless, HSV-1 gene expression and replication remained sensitive to Sp1 expression levels in the absence of ICP0, indicating that Sp1 may be relevant even in contexts where transcription is only partially and not fully inhibited.

### Sp1 can promote virus gene transcription during IFN-mediated restriction of WT virus

**Fig 4:**
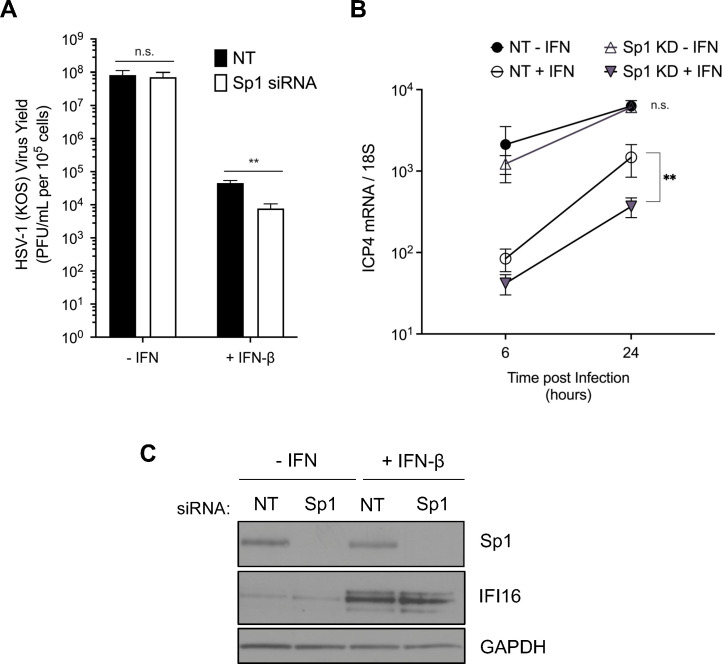
Sp1 promotes WT virus gene transcription to counter IFN-mediated restriction. (**A**) Viral yields were determined following WT virus (MOI = 1, 16 hpi) infection of control or Sp1-depleted HFFs that were untreated or pre-treated with IFN-β (1,000 IU/mL) 1 d prior to infection. (**B**) IE (*ICP4*) gene transcript levels were determined by RT-qPCR analysis following WT virus infection MO1 = 1 of IFN-β pre-treated control and Sp1-depleted fibroblasts. (**C**) Immunoblot analysis of Sp1 and IFI16 levels in pre-treated control and Sp1-depleted HFFs. Statistical significance was determined in panels A and B by two-way ANOVA followed by Tukey’s multiple comparison test. Mean ± S.D. (*error bars*), *P < 0.05, **P < 0.01.

Interferon (IFN) restriction of WT HSV-1 is a key component of immune control during infection and is mediated, at least in part, by the inhibition of IE gene expression in the nucleus (37–40). Given the limited expression and/or activity of virus-encoded transactivators following IFN stimulation, we hypothesized that Sp1 may contribute to viral evasion of this host innate immune response by promoting continued HSV-1 gene transcription in IFN-stimulated cells. To test this, we pre-treated fibroblasts with IFN-β (1,000 U/mL) 24 h prior to infection and compared viral replication and transcript levels in control and Sp1-depleted cells. In the absence of IFN treatment, we did not observe any effect of Sp1 depletion on viral yields during WT HSV-1 infection or on *ICP4* gene transcript levels, as expected (Fig. 4A and B). However, in IFN-β-treated cells, we observed a significant, sixfold reduction in total WT HSV-1 yields when Sp1 was depleted (Fig. 4A). *ICP4* gene transcript levels were similarly reduced fourfold in Sp1-depleted HFFs relative to control cells following IFN treatment (Fig. 4B). Importantly, Sp1 depletion did not alter IFN signaling responses in treated HFFs, as the interferon-stimulated gene IFI16 was induced equivalently in control and Sp1-depleted cells (Fig. 4C). Sp1 can thus promote the increased transcription and replication of WT virus when IFN-induced defenses are activated and the co-opting of the Sp1 transcription factor by HSV-1 may be one additional strategy employed by the virus to evade restriction by the host cell.

### Limited expression of Sp1-family proteins in human iPSC-derived sensory neurons

**Fig 5:**
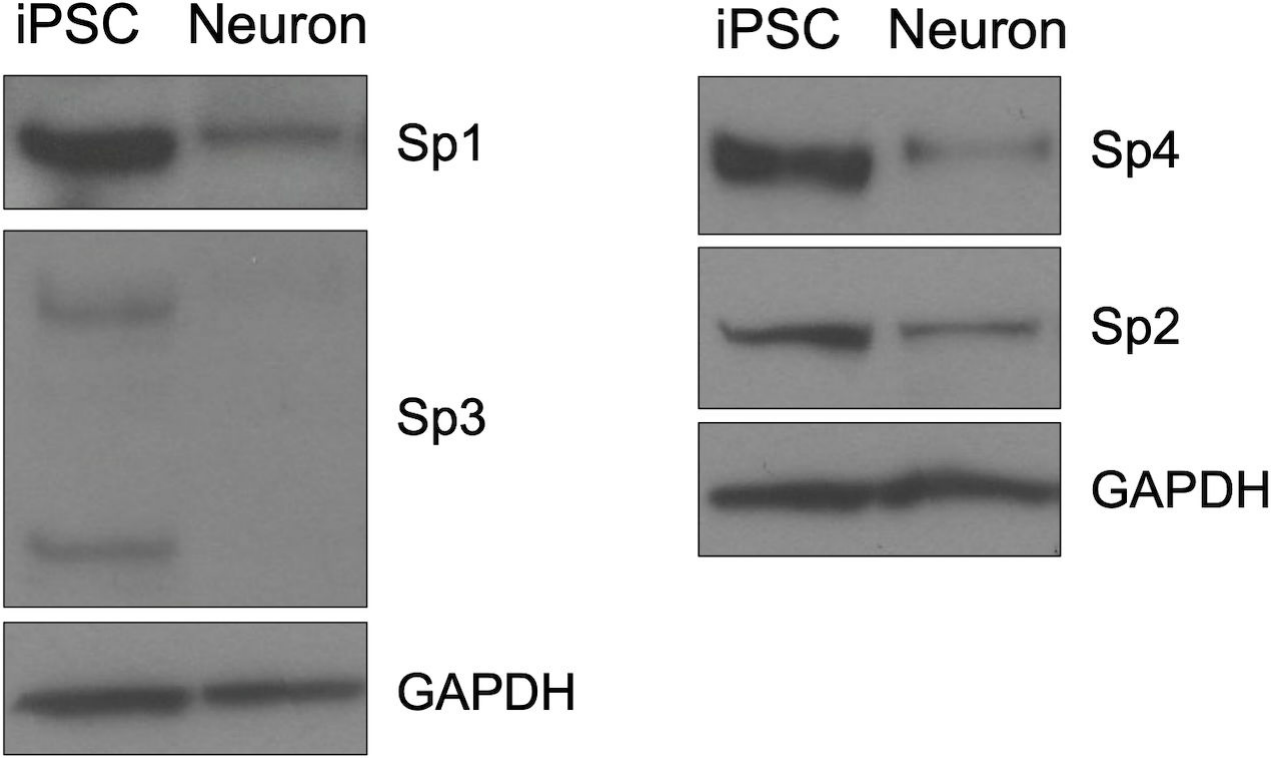
Limited expression of Sp1-family proteins in neurons. Immunoblot analysis of Sp1-family protein expression in lysates from undifferentiated induced pluripotent stem cells (iPSCs) or iPSC-derived sensory neurons.

A limited capacity for viral transcription can determine the outcome of HSV-1 infection when the virus is confronted with activated host defenses, as discussed above, as well as during neuronal infection. Limited VP16 activity at the onset of neuronal infection can promote the establishment of latency, and subsequent reactivation from latency is restricted in the absence of VP16 and ICP0 (5). Our results thus far indicate that Sp1 may enable continued transcription in the absence of these viral transactivators during lytic infection, but whether Sp1 performs a similar function in human sensory neurons, the site of HSV-1 latent infection, is not known. Sp1 is the prototypical member of a family of cellular transcription factors capable of binding at GC-rich sequences to regulate gene expression (26, 41). This larger Sp1 family of transcription factors includes the paralogous set of Sp1-related genes, Sp1–Sp4, that arose from gene duplication events (41). Sp1–Sp4 have similar glutamine-rich N-terminal activation domains and can bind GC boxes via a C-terminal zinc-finger DNA-binding domain. Like Sp1, Sp2 and Sp3 are both ubiquitously expressed, and Sp4 is reported to be particularly enriched in neurons (41). We hypothesized that the reduced activity of virus-encoded transactivators in neurons during latent infection may be compounded by limited expression in neurons of the cellular transcription factors, like Sp1, that could otherwise help maintain ongoing viral gene transcription. We thus compared the expression of Sp1-family proteins in undifferentiated induced pluripotent stem cells (iPSCs) and differentiated iPSC-derived sensory neurons (42), which show the expression of sensory neuron markers (H. S. Oh, S.-F. Chou, P. Raja, J. Shim, B. Das, J. S. Lee, P. Canova, N. Yang, A. Ng, G. A. Church, I. Chiu, C. Woolf, D. M. Coen, D. A. Leib, D. M. Knipe, in preparation). In the sensory neurons, expression of each of the Sp-family proteins was reduced compared to the iPSCs (Fig. 5). This limited expression of Sp1-family proteins may further compromise IE and E gene transcription in neurons and, accordingly, may be contributing to the establishment and/or maintenance of latency during HSV-1 neuronal infection.

## DISCUSSION

**Fig 6:**
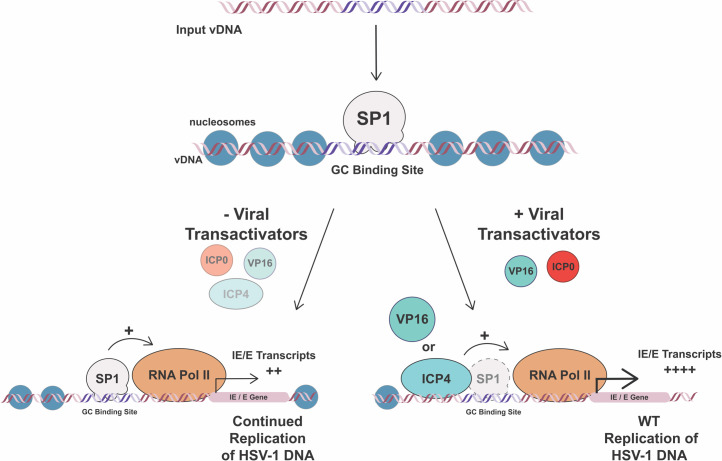
Model: Sp1 facilitates continued HSV-1 gene expression in the absence of key viral transactivators. We observed that Sp1 contributed to continued transcription of viral IE and E gene products when activity of the viral transactivators VP16 and ICP0 was limited. We propose that, when viral transactivators are absent, Sp1 acts to recruit cellular RNA Pol II transcriptional machinery to viral gene promoters to drive continued viral gene expression. Sp1 binding, we predict, may also enhance transcription by reducing levels of restrictive chromatin at these loci, either by directly interfering with nucleosome assembly or via known interactions with cellular chromatin-modifying proteins like p300. VP16 and ICP4 can each act to recruit RNA Pol II to viral gene promoters and VP16 and ICP0 can also facilitate chromatin removal from HSV-1 DNA. When these viral transactivators are present, these activities can ensure a sufficiently high level of viral gene transcription to make Sp1 activity redundant. We hypothesize that HSV-1 maintains the capacity for Sp1 binding to ensure efficient viral gene transcription and productive replication can occur in cellular contexts and environments in which the expression of VP16, ICP4, and/or ICP0 is limited.

A key feature of herpesvirus productive replication is the progression through the lytic gene cascade. The efficiency with which virus gene transcription is initiated and maintained dictates the outcome of HSV-1 infection and is influenced by the activity of both virus and host-cell proteins. In this study, we identified a role for the cellular transcription factor Sp1 in providing an alternative, “back-up” mechanism for viral gene activation during HSV infection (Fig. 6). We determined that Sp1 can promote HSV-1 transcription in conditions or contexts in which the activity or expression of viral transactivators like VP16 or ICP0 is limited. We propose that Sp1 enables continued transcription of viral genes by facilitating access of RNA Pol II and other viral and cellular transactivating proteins to the viral IE and E gene promoters, following Sp1 binding to the GC boxes maintained in these promoters. In the absence of virus-encoded transactivators, Sp1-mediated enhancement of viral transcription is sufficient to stimulate a low level of viral gene expression that can drive continuing viral replication. When viral transactivators are present, however, Sp1 activity becomes redundant as viral proteins instead drive robust RNA Pol II-mediated transcription independently of Sp1. Accordingly, maintenance of Sp1-binding sites in viral gene promoters can potentially enable Sp1 to be co-opted and support ongoing viral gene transcription under adverse conditions in which virus replication would typically be constrained. In support of this model, we found that Sp1 promoted virus gene transcription and contributed to continued HSV-1 replication in IFN-treated fibroblasts and may therefore be relevant to HSV-1 immune evasion. We also observed reduced expression of Sp1 and related Sp1-family member proteins in differentiated sensory neurons, suggesting that limited Sp1 expression may also factor in latency establishment and/or reactivation.

Our observations are generally consistent with findings obtained previously measuring transcript levels of the E thymidine kinase (*tk*) gene following deletion of the two Sp1 binding sites in the *tk* promoter (29, 30). Loss of these Sp1-binding sites dramatically reduced *tk* transcript levels in the absence of ICP4 expression (~20-fold), but only modestly (2- to 3-fold) when ICP4 was present (30). We similarly observed that the impact of Sp1 depletion was more pronounced following infection with VP16- or ICP0-deficient viruses despite a relatively modest effect of Sp1 depletion on WT virus gene transcription and expression as well as yields at early times post infection. Of note, with our depletion-based approach, our results specifically implicate Sp1 in this phenotype rather than Sp1-site-binding proteins more generally. However, the findings from these earlier studies are consistent with the observations made following Sp1 depletion and, thus, lend support to our model in which Sp1 activity is most relevant to viral gene expression when virus-encoded transactivators are absent or limited and in which Sp1 may thus facilitate an alternative back-up mechanism for regulating viral gene expression.

What other cellular transcription factors may also be involved in this regulation remains to be determined. Sp1 is one member of the Sp1-like and Krüppel-like superfamily of transcription factors (26). Different Sp1/KLF-family members can positively or negatively regulate transcription upon binding at GC boxes, with activity potentially varying depending on signaling or other regulatory mechanisms acting within the cell as well as by cell type. Combined effects of these transcription factors can potentially result in additive, synergistic, or competitive regulation of promoter activity downstream of these binding sites. Untangling the roles of all potential binding proteins at these Sp1-binding sites is beyond the scope of this study, but it is worth noting that multiple proteins beyond Sp1 likely act at these sites to regulate transcription and that this area of study is worth pursuing further.

In neurons, as during an activated cellular IFN response, these Sp1-binding sites may provide a mechanism for the virus to co-opt cellular proteins to stimulate viral transcription when viral protein activities are constrained. Compromised activity of the VP16 transactivation complex and reduced expression of cellular transcription factors like Foxc1 have been implicated previously in HSV-1 latency establishment and maintenance (42–44). The reduced expression of Sp1 and Sp1-family proteins observed in our neuronal cell-culture system suggests that reduced expression of these proteins may similarly contribute to promoting an HSV-1 latent state in sensory neurons. Recent studies have implicated transcription factor binding at Sp1 sites in the induction of IE gene expression during stress-induced reactivation events as well (32, 45). It will be worthwhile to investigate whether the expression of Sp1-family proteins and other transcription factors capable of binding these sites are specifically altered following different reactivation stimuli and to test whether increased activity of these factors can contribute to latency establishment and/or reactivation in differentiated human sensory neurons.

Additionally, other DNA viruses, including other herpesviruses like human cytomegalovirus (HCMV) and varicella-zoster virus (VZV), are known to have Sp1-binding sites and/or be regulated by Sp1 and Sp1 family transcription factors (46–52). The findings in this study may thus extend to DNA viruses other than HSV-1 that similarly transcribe and replicate their genomes within the nucleus. As for HSV-1, Sp1 may facilitate the evasion of IFN-induced defenses for these viruses. For the herpesvirus family members containing Sp1-binding sites, Sp1 may also influence entry into or out of latency and may be particularly relevant during VZV latency establishment/reactivation in neurons. Whether Sp1 promotes transcription and replication of these viruses in cells or contexts in which transcription would be limited, as for HSV-1, is not known but would be an interesting area to explore further.

In closing, we propose that the maintenance of Sp1-binding sites in viral gene promoters enables the co-opting of this cellular transcription factor to facilitate viral transcription under conditions or in contexts in which the activity or expression of viral transactivators and other viral gene products is limited, as when the virus is confronted with an activated immune response or during virus infection and reactivation in neurons. Future work investigating the functions of Sp1 and the broader Sp1-family transcription factors at these sites could provide additional insights into how the regulation of viral gene transcription is coordinated between virus and host.

## MATERIALS AND METHODS

### Cell culture

Human foreskin fibroblasts (HFFs; CRL-1635), U2OS (HTB-96), and Vero cells (CCL-81) were obtained from the American Type Culture Collection (ATCC). HFFs were cultured in Dulbecco’s modified Eagle medium (DMEM; Corning) containing 10% heat-inactivated fetal bovine serum (FBS; Gibco) and penicillin-streptomycin (Pen-Strep; Gibco). U2OS and Vero cells were cultured in DMEM supplemented with 5% heat-inactivated bovine calf serum (BCS; Gibco), 5% heat-inactivated FBS, and Pen-Strep. Human-inducible neurogenin3 iPSCs (iNGN3 iPSCs) (53) were seeded and differentiated as described previously, with slight modifications (42). The iNGN3 cells were treated with small molecules (LDN193189, SB-431542, CHIR99021, DAPT, and SU5402) to enhance sensory neuron differentiation (54, 55). All cell lines were grown and maintained at 37°C with 5% CO_2_.

### Viruses and infections

WT HSV-1 strain KOS (56, 57) stocks were propagated in Vero cells, and both stock and virus yield experiment titrations were performed on Vero cells. Both the KOS-derived VP16 activation domain deletion mutant RP5 and the rescued virus RP5R (35) as well as the KOS-derived ICP0-null virus mutant 7134 and the rescued virus 7134R (36) were propagated and titrated in parallel on U2OS cells. Virus stock titers and yield titers were determined by plaque assay following standard procedures (58). Evenly distributed cell monolayers were incubated with virus diluted in phosphate-buffered saline (PBS)-ABC supplemented with 1% heat-inactivated BCS and 0.1% glucose for 1 h in a shaking 37°C incubator. Following removal of the virus inoculum, cells were maintained in DMEM supplemented with 1% heat-inactivated BCS and Pen-Strep at 37°C until the indicated time of harvest.

### siRNA-mediated protein depletion

A non-targeting negative control siRNA pool (D-001810-20) and individual siRNAs targeting Sp1 (J-026959-06-0005, J-026959-08-0005) were purchased from Dharmacon (ON-TARGETplus) and introduced into fibroblasts by transfection, using Lipofectamine RNAiMAX (Invitrogen) according to the manufacturer’s protocol. HFF monolayers were transfected with a final concentration of 10 nM double-stranded siRNAs per well in a 12-well plate before trypsinization and reseeding to a density of 1 × 10^5^ cells per well before harvesting or infection at 72 h post transfection.

### Isolation and quantification of total cellular RNA by quantitative PCR

Viral RNA was quantified as described previously (40). Total cellular RNA was isolated using the RNeasy Mini Kit (Qiagen) and quantified with a NanoDrop spectrophotometer (Thermo Scientific). Purified RNA was treated with DNase (DNA-free, Ambion) prior to reverse transcription with the high-capacity cDNA reverse transcription kit (Applied Biosystems). Relative cDNA levels were measured from duplicate samples using the specific primers (Integrated DNA Technologies) listed in Table 1 using the FAST SYBR green master mix and the 7500 Fast real-time PCR system (Applied Biosystems). Standard curves generated from 10-fold serial dilutions of cDNA prepared from WT KOS- infected HFFs were used for quantification.

**TABLE 1:** Primer sequences for qRT-PCR and qPCR

Target	Sequence (5' → 3')	Reference
qRT-PCR (cDNA)		
18S forward	GCCGCTAGAGGTGAAATTCTTG	(59)
18S reverse	CTTTCGCTCTGGTCCGTCTT	(59)
ICP4 forward	CGGTGATGAAGGAGCTGCTGTTGC	(60)
ICP4 reverse	CTGATCACGCGGCTGCTGTACA	(60)
ICP27 forward	AGACGCCTCGTCCGACGGA	(60)
ICP27 reverse	GAGGCGCGACCACACACTGT	(60)
ICP8 forward	CATCAGCTGCTCCACCTCGCG	(60)
ICP8 reverse	GCAGTACGTGGACCAGGCGGT	(60)
IFI16 forward	ACTGAGTACAACAAAGCCATTTGA	(61)
IFI16 reverse	TTGTGACATTGTCCTGTCCCCAC	(61)
PML forward	GGACCCTATTGACGTTGACC	(62)
PML reverse	TTGATGGAGAAGGCGTACAC	(62)

### Immunoblotting

Immunoblot protein analysis was conducted as described previously (40). Whole-cell lysates were harvested using 1× NuPAGE lithium dodecyl sulfate (LDS) sample buffer (Invitrogen) containing 5% 2-mercaptoethanol and a protease and phosphatase inhibitor cocktail (Halt; Thermo Scientific). Proteins were separated using 4%–12% NuPAGE Bis-Tris polyacrylamide gels (Invitrogen) and transferred to nitrocellulose (Bio-Rad) or polyvinylidene difluoride (Bio-Rad) membranes. Membranes were blocked using Odyssey blocking buffer (LI-COR) or a solution of 5% (wt/vol) nonfat milk in PBS containing 0.1% Tween 20 (PBS-T) prior to an overnight incubation at 4°C with primary antibody. Membranes were washed with PBS-T before secondary antibody incubation at room temperature. The primary and secondary antibodies used are listed in Table 2. Immobilon Classico or Forte Western HRP substrates (Millipore) and X-ray film (HyBlot CL; Denville) were used to detect horseradish peroxidase signal. Fluorescent-labeled antibody signal was detected using the Odyssey CLx Infrared Imaging System (LI-COR).

**TABLE 2:** Antibodies for immunoblot experiments

Antibody target	Source	Identifier
GAPDH	Abcam	ab8245
ICP0	East Coast Bio	H1A027
ICP4 (58S)	(63)	
ICP27	Abcam	ab31631
ICP8 (3-83)	(64)	
Sp1	Abcam	ab124804
Sp2	Abcam	ab229468
Sp3	Santa Cruz	sc-365220
Sp4	Santa Cruz	sc-390124
Mouse IgG, HRP-conjugated (secondary antibody)	Cell Signaling	7076S
Rabbit IgG, HRP-conjugated (secondary antibody)	Cell Signaling	7074S
Mouse IgG, IRDye 680RD (secondary antibody)	LICOR	926–68070
Rabbit IgG, IRDye 800CW (secondary antibody)	LICOR	926–32211

### Interferon treatment

HFFs were untreated (-IFN) or pre-treated for 20 h with IFN-β (1,000 U/mL; R&D Systems) in DMEM containing 10% FBS and Pen-Strep and washed once with PBS-ABC (PBS with MgCl_2_ and CaCl_2_) prior to infection. No IFN was added during infection or subsequent incubations.

### Statistical analyses

Data analysis was performed using GraphPad Prism version 9.0. Means across multiple groups were analyzed by ANOVA and relevant *post-hoc* tests, and statistical significance is indicated when relevant, with *P < 0.05; **P < 0.01; ns, not significant.

## SUPPLEMENTAL MATERIAL

Supplemental Figures mbio.03479-23-s0001.pdf Figures S1-S4.

ASM does not own the copyrights to Supplemental Material that may be linked to, or accessed through, an article. The authors have granted ASM a non-exclusive, world-wide license to publish the Supplemental Material files. Please contact the corresponding author directly for reuse.
