# Supplementary material for: Sp1 facilitates continued HSV-1 gene expression in the absence of key viral transactivators
Source: mBio. 2024 Feb 13;15(3):e03479-23. doi: 10.1128/mbio.03479-23 (PMC10936440; doi:10.1128/mbio.03479-23)
Supplement: Supplemental Figures — Figures S1-S4. [file mbio.03479-23-s0001.pdf]

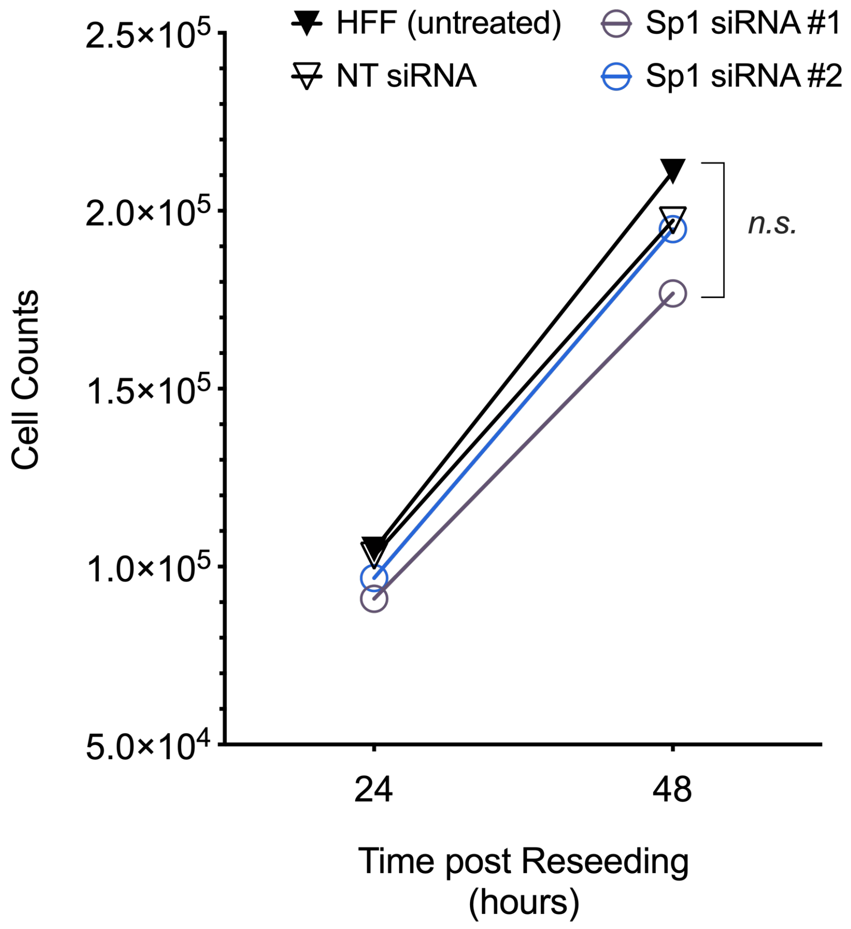

Supplementary Figure 1. Sp1 depletion by individual siRNAs does not inhibit cell growth in fibroblasts.

Supplementary Figure 1. Sp1 depletion by individual siRNAs does not inhibit cell growth in fibroblasts.

Cell growth of HFFs treated with non-targeting (NT) control siRNAs or individual Sp1-specific siRNAs was determined by counting siRNA-treated cells after re-seeding at 48 h post transfection. Statistical significance was determined by two-way ANOVA followed by Tukey's multiple comparison test. Means from four replicates shown, * $P < 0.05$, ** $P < 0.01$.

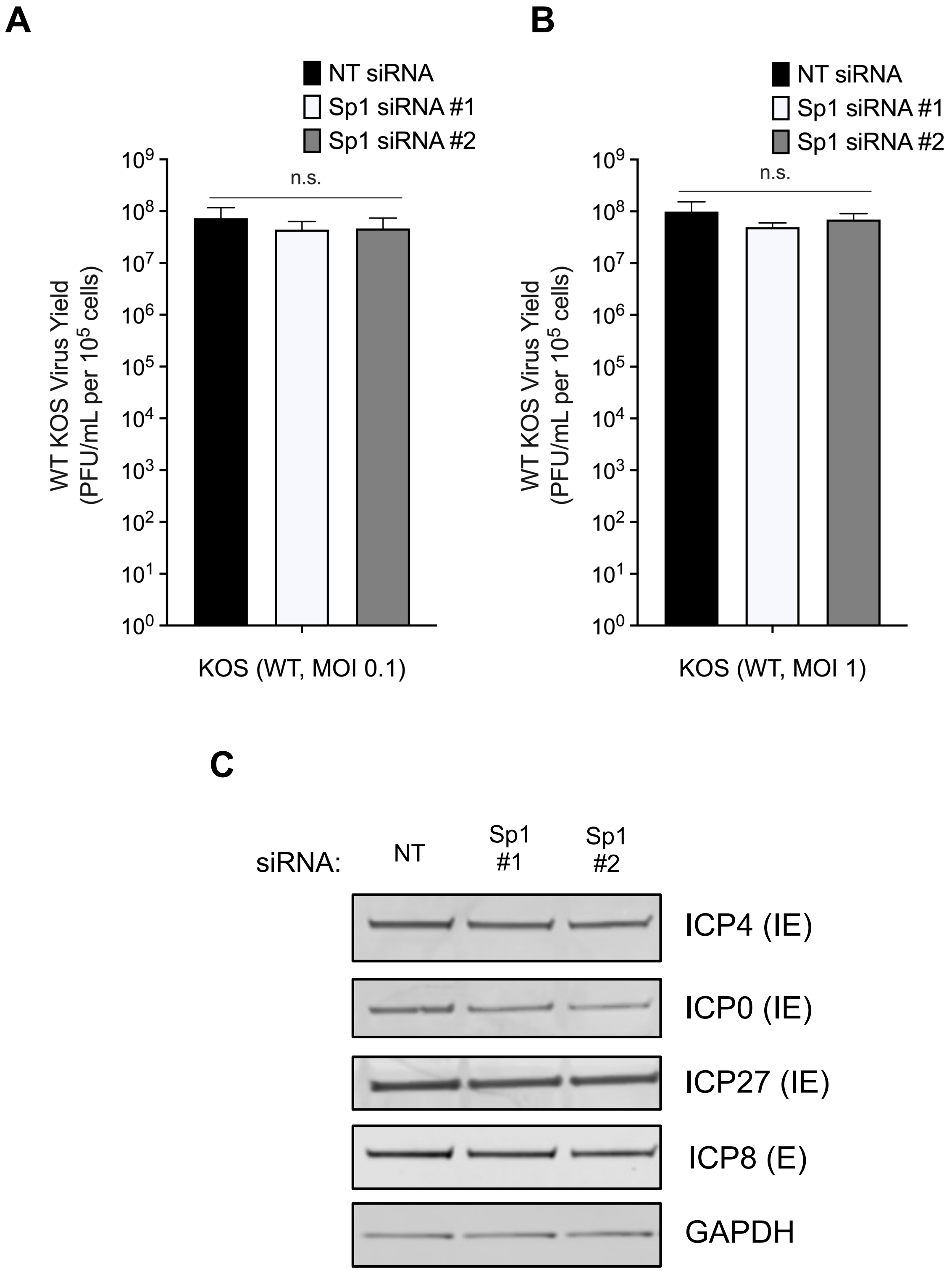

Supplementary Figure 2. Individual Sp1-specific siRNAs have equivalent effects on WT HSV-1 replication and gene expression.

Supplementary Figure 2. Individual Sp1-specific siRNAs have equivalent effects on WT HSV-1 replication and gene expression. (A) Viral yields were determined following infection of HFFs transfected with the control non-targeting (NT) siRNA or the individual Sp1-specific siRNAs with WT KOS (MOI=0.1, 48 hpi) or (B) at an MOI of 1 for 24 h. (C) Immunoblot analysis of viral protein expression following WT infection of NT or Sp1 siRNA-treated HFFs (MOI=3, 8 hpi). Statistical significance was determined in panels A and B by two-way ANOVA followed by Tukey's multiple comparison test. Mean ± S.D. (*error bars*), * $P < 0.05$, ** $P < 0.01$.

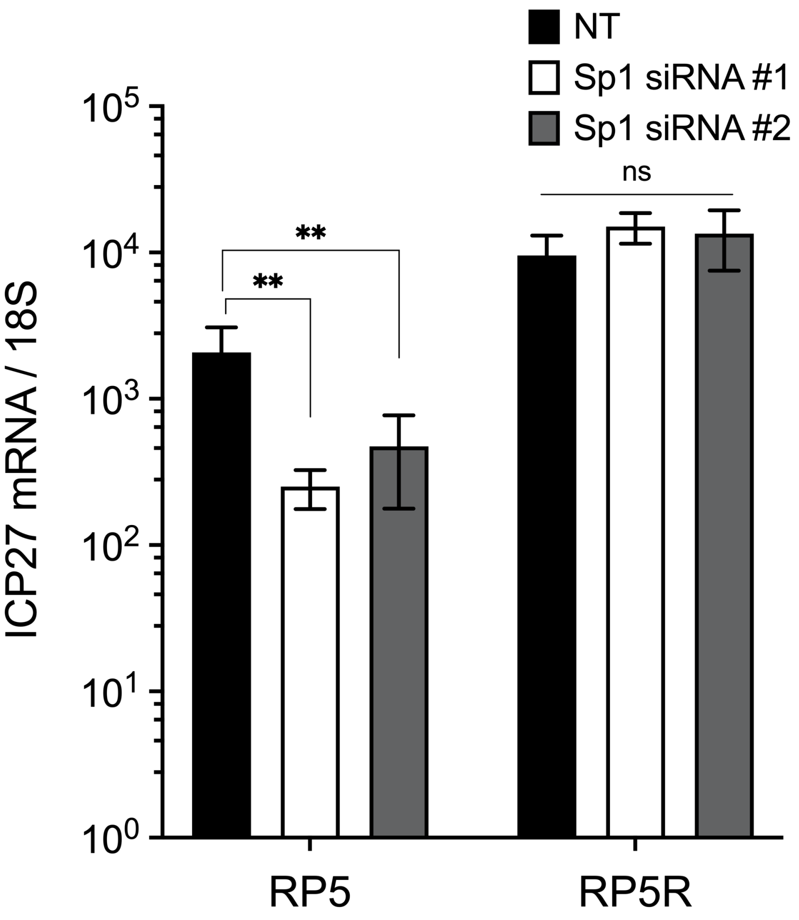

Supplementary Figure 3. Individual Sp1-specific siRNAs reduce virus transcript levels comparably in the absence of functional VP16.

Supplementary Figure 3. Individual Sp1-specific siRNAs reduce virus transcript levels comparably in the absence of functional VP16. IE transcript levels were determined by qRT-PCR and compared following infection of HFFs treated with NT or individual Sp1-specific siRNAs with either the RP5 VP16-mutant virus or the wild-type-equivalent rescued virus RP5R (MOI=10, 6 hpi). Statistical significance was determined by two-way ANOVA followed by Tukey's multiple comparison test. Mean ± S.D. (*error bars*), * $P < 0.05$, ** $P < 0.01$.

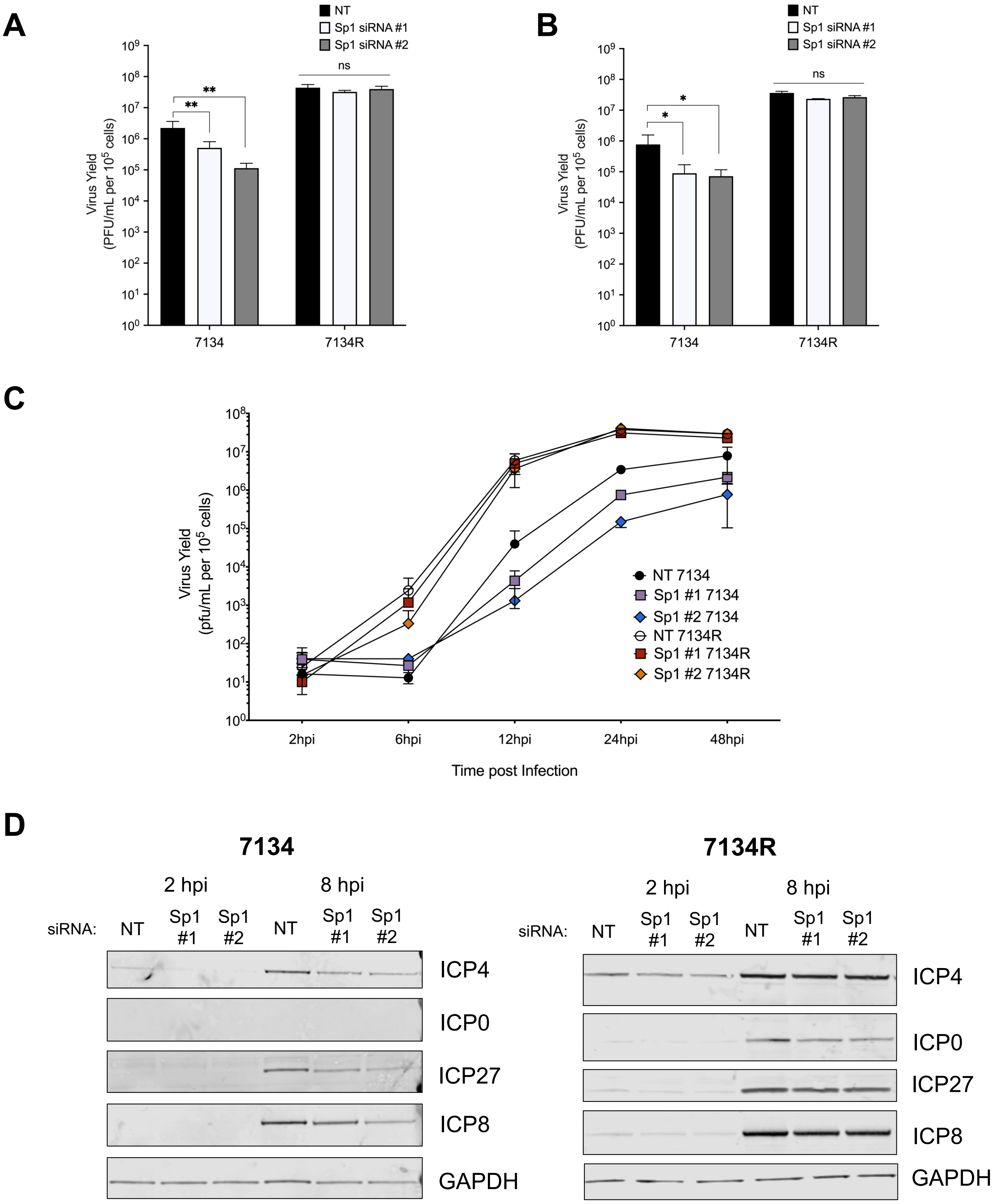

Supplementary Figure 4. Individual Sp1-specific siRNAs reduce virus replication and gene expression during ICP0-null virus infection.

Supplementary Figure 4. Individual Sp1-specific siRNAs reduce virus replication and gene expression during ICP0-null virus infection. (A) Virus yields were determined following infection with 7134 or 7134R viruses (MOI=1, 24 hpi) in Sp1-depleted HFFs. (B) Viral yields following infection of Sp1-depleted HFFs with 7134 and 7134R viruses at MOI of 0.1 for 48 h. (C) Viral yield time course in HFFs treated with non-targeting (NT) or individual Sp1-specific siRNAs after infection with the ICP0-null virus 7134 viruses or the ICP0-positive rescued virus 7134R (MOI=1). (D) Immunoblot analysis of IE and E viral gene expression following Sp1 depletion with individual siRNAs and 7134 or 7134R viral infection (MOI=3) in HFFs. Statistical significance in panels A and B was determined by two-way ANOVA followed by Tukey's multiple comparison test. Mean ± S.D. (*error bars*), * $P < 0.05$, ** $P < 0.01$.
